# Can templates-for-rejection suppress real-world affective objects in visual search?

**DOI:** 10.3758/s13423-023-02410-2

**Published:** 2024-02-05

**Authors:** Chris R. H. Brown, Nazanin Derakshan

**Affiliations:** 1 School of Psychology, University of Roehampton, Whitelands Campus, Holybourne Avenue, London, SW15 4JD UK; 2 School of Psychology and Clinical Language Sciences, University of Reading, Reading, RG6 6ET UK

**Keywords:** Templates-for-rejection, Negative templates, Attentional bias, Cognitive and attentional control

## Abstract

Previous evidence has suggested that feature-based templates-for-rejection can be maintained in working memory to suppress matching features in the environment. Currently, this effect has only been demonstrated using abstract neutral shapes, meaning that it is unclear whether this generalizes to real-world images, including aversive stimuli. In the current investigation, participants searched amongst an array of real-world objects for a target, after being precued with either a distractor template, target template, or a no template baseline. In Experiment 1, where both distractor and target template cues were presented randomly on a trial-by-trial basis, there was moderate evidence of increased capture by aversive distractors after the distractor template cue. In Experiment 2a, however, when distractor templates were the only available cue and more time was given to encode the cue features, there was moderate evidence of effective distractor inhibition for real-world aversive and neutral stimuli. In Experiment 2b, when the task required a slower more effortful comparison of target features to stereotypical object representations, there was weaker evidence of inhibition, though there was still modest evidence suggesting effective inhibition of aversive distractors. A Bayesian meta-analysis revealed that across Experiment 2, aversive distractors showed strong cumulative evidence of effective inhibition, but inconsistent inhibition for neutral distractors. The results are interpreted from a rational search behaviour framework, which suggests that individuals utilize informative cues when they enable the most beneficial strategy and are accessible, and apply these to distractors when they cause sufficient disruption, either to search speed or emotional state.

**Supplementary Information:**

The online version contains supplementary material available at 10.3758/s13423-023-02410-2.

The ability to suppress irrelevant stimuli in the environment is vital for effective goal-pursuit. One possible mechanism of suppression is through a deliberate template-for-rejection (aka ‘negative template’), whereby a cued distractor representation held in visual working memory (VWM) can be used to inhibit matching features. Earlier evidence reveals that after distractor features are cued, participants are faster to identify the target compared with when they are given no information about the upcoming distractor (Arita et al., 2012). Evidence in support of enhanced inhibition shows that participants are slower to identify features which were earlier embedded in a cued distractor (Chang & Egeth, 2019). Additionally, eye-tracking has revealed that a cued template-for-rejection results in fewer saccades towards a distractor, relative to when no prior information is given (Zhang et al., 2022).

Much of this research, however, has focused on simple coloured shapes, suggesting that the efficacy of this flexible inhibitory mechanism for real-world stimuli, which are multifaceted and hold associated value, has not been fully investigated. It is possible that individuals may be more motivated to ignore these stimuli, especially emotionally aversive images (Armstrong et al., 2014). Though evolutionarily adaptive to detect aversive stimuli, once an individual is aware that this is not a direct threat, the ability to suppress irrelevant aversive images is beneficial, as evidence suggests they disrupt VWM functioning (Figueira et al., 2017), and long-term can cause anxiety (Van Bockstaele et al., 2014).

Thus far, only one study conducted by Kennedy et al. (2018) has explored the influence of deliberate cued suppression on photorealistic affective stimuli. In this study, participants completed an RSVP task with erotic or threat-related distractors. In mini-blocks of 8–12 trials, participants were cued with an affective distractor category (e.g., “ignore gruesome”), which resulted in more accurate target identification. It is not clear, however, whether this result is due to feature specific templates-for-rejection held in VWM, as the warning benefit did not differ depending on the specific cued feature (i.e., “ignore gruesome” versus “ignore graphic”). Additionally, the prewarned trials were presented in mini-blocks, preventing inference about the trial-by-trial flexibility of the VWM representation. It is possible that effects only occur after repeated trials, due to a learning mechanism, rather than a VWM-based template (Muhl-Richardson et al., 2022; Schmidts et al., 2020).

## Current investigation

In three experiments, we examined whether templates-for-rejection could effectively suppress images of real-world stimuli when varied on a trial-by-trial basis. To assess the proposed mechanisms with realistic objects, participants searched amongst everyday objects for a uniquely oriented target (Experiments 1a and 2a), or as the only object inverted from its usual position (Experiment 2b), thus requiring a more conceptual search criteria based on comparison to long-term memory.

In Experiment 1, participants were briefly cued with either the distractor features (distractor template), target features (target template), or a non-informative cue (no template), presented in a random order; whilst in Experiment 2a/2b, we only presented the distractor template and no template conditions. Across experiments we presented real-world neutral and aversive distractors, which consisted of photographic images of healthy or severely injured hands, matched for size and shape. These were presented alongside a no-distractor baseline, consisting of a low-salience placeholder. As a further control condition in Experiment 2a/2b, we presented a salient coloured circle distractor, to isolate distractor effects independent of salience.

Whilst there have been some failures to replicate the template-for-rejection effect (Beck et al., 2018; Berggren & Eimer, 2021), it does appear that under some conditions templates-for-rejection can effectively inhibit distractor features. Specifically, when the strategy is beneficial in increasing search efficiency, due to the task being perceptually demanding (Conci et al., 2019), the distractor appearing as sufficiently salient relative to the target (Kerzel & Huynh Cong, 2022), and there is sufficient time to prepare for stimulus onset (Tanda & Kawahara, 2019). Therefore, to maximize the chances that the template-for-rejection would be effective, we presented the target amongst a complex array of multifaceted and equally salient coloured objects, with a long cue-array interval. Additionally, within Experiment 2a/2b, we also increased the distractor cue presentation time to facilitate the encoding of its features (Gibson & Bryant, 2005).

There are three possible outcomes from the current study, the first being that templates-for-rejection are effective at suppressing real-world image distractors, especially aversive stimuli which can elicit an avoidance response after initial exposure (Armstrong et al., 2014). If true, the distractor cost, as measured by subtracting the no distractor condition reaction time (RT) from the distractor present RT, will be lower in the distractor template condition relative to the no template condition. Alternatively, cueing real-world distractor imagery could result in a paradoxical increase in attentional capture (Beck et al., 2018); and this could be exacerbated for aversive distractors, which have been found to disrupt VWM (Figueira et al., 2017). The final possibility is that templates-for-rejection are simply ineffective at suppressing photorealistic imagery, but do not cause heightened capture, resulting in no difference between the distractor template and no template trials. To assess the evidence for and against these competing hypotheses, we computed multiple Bayesian pairwise comparisons with different priors reflecting these opposing expectations.

## Methods

### Participants

#### Experiment 1

Initially, 41 participants were recruited, though one participant was excluded for falling outside the 18–40 age range inclusion criteria. The final sample included 26 females and 14 males, the mean age of which was 25 years (*SD* = 4.88). The sample was determined by the maximum number of participants that could be recruited within a single academic term. The final sample was powered to detect an effect size of *d*_z_ = .58 (β = .95, α = .05), making it sufficient to detect prior templates-for-rejection effects in similar task designs (i.e., Arita et al., 2012; Conci et al., 2019). Participation was in exchange for course credit. Participants completed the experiment on an 18-inch Mitsubishi monitor with a screen resolution of 1,152 × 864 and a refresh rate of 75 Hz. Participants viewed the screen from a distance of 60cm maintained with a chinrest.

#### Experiment 2a

For Experiment 2a, 32 participants were initially recruited, though two were excluded due to poor performance (accuracy <60%). The sample was powered to detect prior evidence of templates-for-rejection effects (e.g., *d*_*z*_ = 1.02 from Conci et al., 2019, required *N* = 15, α = .05, β = .95), though a larger sample of 30 was recruited in line with central limit theorem to ensure normality (Kwak & Kim, 2017). The final sample consisted of 22 female and eight male participants, with the average age of 20.60 years (*SD* = 3.15). Participants in both Experiment 2a and 2b completed the experiment on an HP EliteDisplay E221c 21.5-inch monitor with a screen resolution of 1,920 × 1,080 resolution and a refresh rate of 60 Hz. Participants viewed the screen from a 60-cm distance, maintained with a chin rest.

#### Experiment 2b

Initially, as preregistered (osf.io/hv42u), 54 participants were recruited, though four were excluded for poor performance on the task (accuracy <60%). The final sample included 43 female and seven male participants, and one participant who preferred not to report their gender. The average age was 20.20 years (*SD* = 2.16).

The large pre-registered sample for Experiment 2b was designed to have a high level of power (α = .05, β = .95) to detect any significant correlation with trait anxiety based on the detected effect in Experiment 1 (*r* = .45). Due, however, to a limited recruitment period, exclusions were not replaced. The final sample of 50 participants was well powered to detect expected within-subjects effects.

### Stimuli and materials

#### Experiment 1

**Fig. 1:**
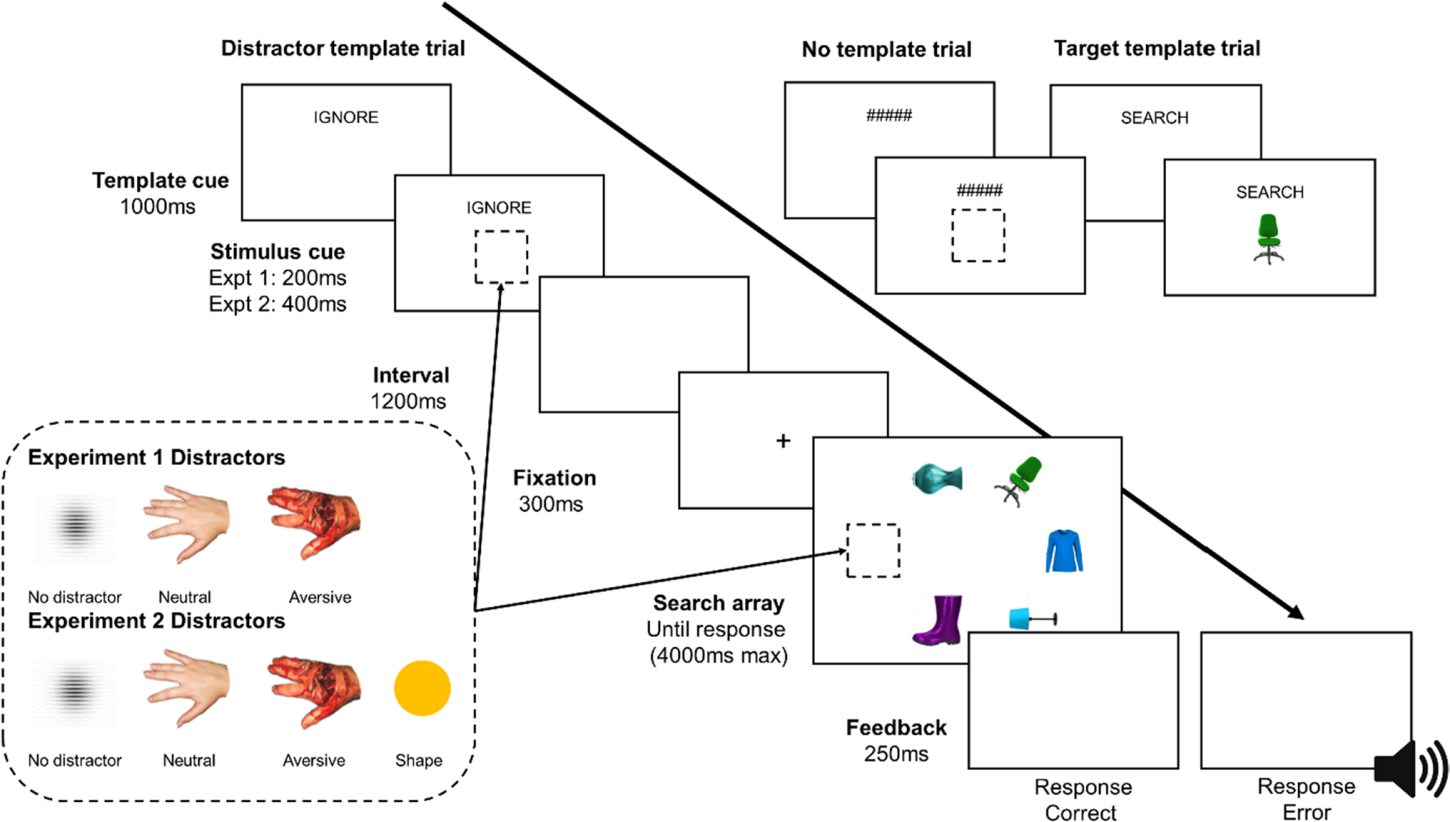
Depiction of a trial sequence for each of the three template conditions: distractor template, target template, and a no template condition. The target template trials were only presented in Experiment 1. In Experiment 1 and 2a the target was always the only stimulus rotated 45° amongst upright and horizontal stimuli (as in figure), in Experiment 2b the target was always the rotated 45° inverted image amongst 45° rotated upright images. Exemplars of all distractor types are presented and labelled with the Experiments they appeared in. (Colour figure online)

In order to create a visually heterogenous stimulus array with real-world objects, we created a novel stimulus set. This consisted of seven object categories, which included multiple exemplars of chairs, cups, lamps, shirts, umbrellas, vases, and shoes. Of each object category there were four different exemplars presented as non-target filler images which populated the array, and a single exemplar which only appeared as the target (see Fig. 1). The exception was the shoe category, which for counterbalancing, only included four non-target exemplars and never appeared as the target. In total there were images of 35 different objects, which were all sourced from Google Images from non-copyrighted sources.

To control for differences in salience and colour across objects, the objects were recoloured in shades of blue, green, yellow, orange, turquois, and purple, to create six different versions of each exemplar. Due to differences in texture and shading within and between objects, a uniform colour was not possible, therefore the colour of each object was visually matched by the authors and checked by another researcher. All images are available via the OSF (https://osf.io/nrk7w/?view_only=aa31bf3a33914a98a5a6ca74794b1ed3). In total, there were 36 different target exemplars (6 object types × 1 exemplar per object × 6 colours), and 210 filler exemplars (7 object types × 5 exemplars per object × 6 colours).

The distractor images were sourced from Google images and consisted of images of healthy and injured hands.^1^ These were selected due to their ability to depict strong affective information and match for visual complexity. The content was similar to the images of injury from existing stimulus sets (e.g., International Affective Picture System; Lang et al., 1997), though novel images were sourced to ensure similar resolution and matching across aversive and neutral distractor types. For the aversive distractors, twelve hand images depicting mutilation from frostbite, severe burns, lacerations, and abrasions were selected. Twelve neutral hand images were also selected, these consisted of hands with similar outlines to the aversive set and with relaxed and inactive poses so as not to confer any social cues.

For the no distractor condition, a black and white 90° Gabor-patch placeholder was presented in place of a filler or distractor image. All images had their original background erased and were resized to fit within a 2.3° square. Across the experiment the default background colour was white.

#### Experiment 2a and 2b

To assess the efficacy of templates-for-rejection with abstract shape distractors as used in previous investigations, a salient orange coloured circle distractor was also included (RGB colour code: 255, 127, 39). The inclusion of the orange distractor resulted in the subsequent change to the colours the target and filler objects could appear in due to feature overlap, thus the colours yellow and orange were replaced with the colours light grey and black. In order not to incidentally cue attention to the grey features in the array, the grey square on the no-template trials was replaced by a dashed outline of a square.

## Questionnaires

### State-Trait Anxiety Inventory (STAI; Spielberger et al., 1983)

The STAI is a widely used well-known 40-item questionnaire with 20 items assessing current levels of state anxiety, and 20 items measuring long-term trait anxiety.

### Procedure

#### Experiment 1

Prior to the task, participants were given the option to view an example injured hand image which was not part of the experimental image set, to ensure fully informed consent. The task was a 3 × 3 within-subjects design with template type (target template, distractor template, no template) and distractor type (aversive distractor, neutral distractor, no distractor) as conditions. All conditions were presented across three blocks of 108 trials (overall 32 trials per condition), with the trial order randomized.

Each trial began with a template instruction cue lasting 1,000 ms, priming participants with which template type they must utilize for that trial. The target template trials were preceded by the text cue “SEARCH,” the distractor template trials were preceded by an “IGNORE” text cue, and the no template trials were preceded by a non-informative “#####” text cue. Immediately after the text template cue, the stimulus information was presented below the text cue for 200 ms whilst the text cue remained on the screen. In all conditions, the image was presented as a 3° × 3°sized stimulus. In the target template condition, the stimulus was the target that participants had to identify the orientation of, though it appeared vertically to not prime the response. In the distractor template condition, the stimulus was the distractor image, and in the no template condition, a non-informative grey square (RGB colour codes: 192,192,192) was presented.

Following the cue, there was an interstimulus interval of 1,200 ms, followed by a 300-ms fixation cross, before the visual search array was presented. The array consisted of six equidistant stimuli presented in an imaginary circle around the centre of the screen, with a diameter of 10.1°. One image was always the target, and one the distractor, with the other four random fillers appearing in different colours. All stimuli were presented as 2.3° squares in the array. The target was the only stimulus rotated 45° to the left or right, whilst the distractor was presented upright. Two of the filler images were presented upright, and one rotated 90° to the left, and one 90° to the right. Participants used the left and right arrow keys to indicate which orientation the rotated target image was. They were instructed to respond as fast as possible whilst remaining accurate.

Within each block the template type, distractor type, target orientation, and target object type (i.e., chair, cup, shirt, umbrella, vase), was fully counterbalanced. The target positions were randomly selected each trial, though the target appeared at each of the 6 positions an equal number of times for each template type, distractor type, and response type (i.e., left/right). Similarly, the distractor exemplars were randomly selected, but all appeared an equal number of times in each of the template type conditions. The distractor position was randomly selected each trial, as was the filler colour and objects; though the filler objects and colours were randomly selected such that they never matched the target object category or colour. After the participants’ response or 4,000 ms had passed, a 250-ms feedback screen was presented with a short beep delivered through headphones on erroneous responses, and a blank screen for correct responses.

Prior to the task, participants completed an 18-trial practice block consisting of six trials for each template type and each type of target object within these conditions. All trials were no distractor condition to prevent extensive learning, and the target colours, positions, and orientation were fully randomized. Once the task was completed, participants completed a computerized version of the STAI using Qualtrics software, before being debriefed. See Supplementary Materials 1 for full analysis of anxiety data.

#### Experiment 2a

The changes made to Experiment 2a were (1) the increase in the cue presentation time from 200 ms to 400 ms to facilitate encoding of the visually complex and varied multidimensional distractor stimuli, based on the design of previous investigations assessing VWM storage of real-world imagery (Hu & Jacobs, 2021; Miuccio et al., 2022); (2) the removal of the target template condition to allow exploration of the template-for-rejection effect isolated from target cueing effects; (3) the addition of a salient non-object shape distractor to isolate the relative effect of templates-for-rejection on real-world objects versus non-object stimuli.

In total, there were 288 trials in the task, with 96 trials across three blocks. Half the trials were cued with the distractor template, and the other half with no template, thus yielding 32 trials in each of the eight conditions.

#### Experiment 2b

The only change to Experiment 2b was the alteration of the target and non-target object orientations. To encourage participants to utilize semantic information in long-term memory to identify the target, as required in real-world visual search, participants were instructed to identify the 45° orientation of the only inverted object. This was presented amongst other 45° rotated upright objects. This would require participants to compare the objects to a stereotypical representation in long-term memory. For the upright non-target stimuli in the array, two were rotated 45° to the left, and two 45° to the right.

### Statistical analysis

The dependent variable for the visual search task was RT for correct trials, trials in which responses were incorrect or were over 2,500 ms or under 100 ms were excluded. In the current investigation we took a Bayesian approach to the analysis, to compare the evidence for and against competing *effective inhibition* versus *increased capture* hypotheses, as well as the null effects for both hypotheses—which is not possible with conventional frequentist statistics (Dienes & Mclatchie, 2018). Exploratory analyses, however, were computed with frequentist statistics due to the lack of prior expectations.

Bayesian analysis is conducted by contrasting an expected effect size for a comparison (prior), based on a specific theory, to the observed value of that comparison in the data (likelihood). Based on the combination of the prior and likelihood, we obtain the posterior probability that the observed evidence supports or contradicts the initial theory (Dienes, 2011, 2014; Wagenmakers et al., 2018). The key statistics computed in this analysis are Bayes factors, which provide a numeric value for whether the observed effect is more likely to reflect evidence for the experimental hypothesis, or whether an effect is more likely to reflect evidence for a null hypothesis. Bayes factors greater than 1 reflect evidence favouring the experimental hypothesis, whilst those less than 1 reflect evidence favouring the null (i.e., no difference).

Though Bayes factors are interpreted as a continuous measure of evidence, rather than using strict cut-off criteria as with *p* values, we utilized classic interpretation guidelines of evidence to assist reporting and interpretation (Jeffreys, 1961; Lee & Wagenmakers, 2014). A value of 1 suggests that any difference is anecdotal and is inconclusive until more data are collected. Whilst values greater than 3 reflect moderate evidence for the experimental hypothesis, and less than .33 reflect moderate evidence for the null hypothesis. The specific parameters for each Bayesian analysis are listed below next to the relevant analysis.

A full frequentist analysis is reported in Supplementary Materials 2, and notable frequentist results are highlighted in the main text. All analyses for Experiment 2b were preregistered prior to data collection (osf.io/hv42u) this preregistered analysis was also applied to Experiment 1 and 2a for consistency. Deviations from the preregistration are noted in the text.

#### Planned Bayesian comparisons

##### Distractor cost effects

In order to gain a clear interpretable measure of change in RT we computed distractor cost scores by subtracting the no distractor RT from both the neutral and aversive distractor RTs, separately. These scores were computed separately for all distractor and template types. If distractor costs were greater than zero this reflected greater interference when the distractor was present versus absent.

To calculate Bayes factors for these two competing hypotheses, we calculated multiple Bayes factors with two different opposing priors using the mean raw RT difference from published data. One prior reflected faster target detection when the distractor was present, versus absent (mean difference = −22 ms), consistent with effective inhibition; and another reflecting slower target detection when the distractor was present (mean difference = 65 ms), reflecting attentional capture. These priors were both taken from Gaspelin et al. (2015; Experiment 1 for capture, and Experiment 3 for inhibition) who found evidence for both suppression and capture by salient distractors in a similar visual search task. These priors were based purely on general inhibition and capture by distractors and were not related to specific templates-for-rejection effects. All Bayesian analyses were calculated with the expected mean effect size (from previous research) set as the prior. This prior was set as a directional half-normal distribution, with the mean expected effect size modelled as a standard deviation centred on zero, reflecting the null. The use of an informed half-normal distribution makes the analysis more sensitive to smaller effects, as they are estimated to be more probable, causing small effects to result in anecdotal evidence rather than strongly favouring the null hypothesis (Dienes, 2014).

##### Template cueing effects

The key comparisons which tested our specific hypotheses were the comparison of the distractor costs between the distractor template condition and the no template condition. If distractor costs were lower in the distractor template condition then this would be evidence that the template-for-rejection was effective at suppressing distractor interference, whilst a higher distractor cost in the distractor template condition would be reflective of counterproductive attentional capture.

To calculate Bayes factors for these two competing hypotheses, we calculated multiple Bayes factors with two different opposing priors using the mean raw RT difference from published data. One prior reflected effective template cued distractor inhibition (−42 ms), and another reflecting template cued attentional capture (40 ms). These priors were both taken from Cunningham and Egeth (2016; Experiment 1, Block 1 for capture, and Block 4 for suppression) who found evidence for both effective inhibition as well as increased capture after using templates-for-rejection in a similar visual search task using coloured letters. To directly compare target and distractor template cueing effects, we retained the same priors when analyzing target template effects. These Bayesian priors were set as a directional half-normal distribution, with the mean expected effect size modelled as a standard deviation centred on zero, reflecting the null. The use of the half-normal distribution was again utilized to be sensitive to small effects to avoid strongly favouring the null hypothesis (Dienes, 2014).

For transparency we also conducted the analysis using a uniformly distributed prior which does not accommodate variation in probable effect sizes and is more data-driven. We note that this reanalysis found stronger evidence for effective inhibition, with the half-normal distribution being relatively conservative (see Table 2 and reanalysis with uniform priors in Supplementary Materials 3 for comparison).

##### Post hoc Bayesian meta-analysis

Within Experiment 2a and 2b, the pattern of results showed some inconsistency despite these experiments being highly similar and only differing in the target’s identifying feature. Therefore, to assess the cumulative evidence within Experiment 2 as a whole, we computed Bayes factors using the posterior estimate of the mean and *SE* of each preregistered distractor template cueing effect from Experiments 2a and 2b, using the metacalculator shinyapp (https://neilmclatchie.shinyapps.io/MetaCalculator/). We first entered the template cueing effect from Experiment 2a as the prior distribution and the same effect from 2b as the likelihood, to produce the posterior mean and *SE* effects. The same priors for the original analysis were used to calculate Bayes factors from this posterior estimate for each distractor.

#### Contrasting specific distractor template effects

##### Aversive versus neutral distractor

To make a direct comparison of whether an observed template-for-rejection effect was stronger for affective or neutral distractors, we compared the template cueing effects between aversive and neutral distractors. A higher score would reflect greater attentional capture by aversive distractors, whilst a negative score would reflect greater inhibition of the aversive distractor. Both the Bayesian priors for effective inhibition (−42 ms) and increased capture (40 ms) were retained for the comparison, based on the logic that the largest possible effect would occur if one distractor resulted in the expected inhibition or capture effect whilst the other was unaffected, resulting in zero template cueing effect.

##### Real-world versus salient shape distractor

In Experiment 2a and 2b, the inclusion of the salient shape distractor allowed the comparison of distractor template cueing effects for real-world versus more conventional salient distractor stimuli. This comparison allowed us to explore whether the observed effective inhibition or increased capture effects were limited to photorealistic stimuli, or generalized to all salient stimuli. The real-world distractor costs were computed by averaging the distractor costs for both neutral and aversive hand distractor stimuli. These were then contrasted with the shape distractor cost in a Bayesian pairwise comparison. Again, both the previous Bayesian priors for effective inhibition (−42 ms) and increased capture (40 ms) were retained for the comparison, based on the same logic as the aversive versus neutral template cueing effect comparison.

### Exploratory block analysis

A follow-up block analysis was also conducted to explore possible differences across the task found in previous research (Cunningham & Egeth, 2016). In Experiment 1 this was investigated using a 2 × 3 × 3 repeated-measures ANOVA which compared the difference in RT on distractor template trials versus no template trials across the three distractor conditions (i.e., no distractor, neutral distractor, aversive distractor) across all three blocks of the task. In Experiment 2a and 2b, the same analysis was repeated with the exception that the shape distractor was added to the analysis, making it a 2 × 4 × 3 repeated-measures ANOVA.

### Accuracy

As RT was the main dependent variable, we did not conduct a Bayesian analysis of accuracy due to no expectations of the specific pattern of results. However, to explore whether there were any differences in accuracy across conditions, we conducted a repeated-measures ANOVA. For Experiment 1 this was a 3 × 3 design, and in Experiment 2a and 2b this was a 2 × 4 design.

## Results

### Distractor cost comparisons

For RT, effect sizes, and Bayes factors, see Table 1. Bayesian analysis of the difference between all distractor types versus the no distractor condition in Experiments 1–2b revealed that there was at least moderate evidence of attentional capture by real-world distractors (BF > 3). The exception was the aversive distractor in the distractor template condition in Experiment 2a, which showed weak anecdotal evidence of capture (BF ~ 1).

**Table 1:** Mean and standard error (*SE*) of reaction time (RT) across all conditions of Experiments 1, 2a, and 2b

	Template	Distractor	Reaction time ms (*SE*)	Accuracy (*SE*)	Distractor cost RT (*SE*)	Distractor cost Cohen’s *d*_*z*_	H_inhibition_ Bayes factor	H_capture_ Bayes factor
Experiment 1	No template	No distractor	889 (22)	.96 (.01)	–	–	–	–
		Aversive	920 (24)	.97 (.01)	31 (11)	.46	**.12**	**19.45**
		Neutral	946 (25)	.95 (.01)	57 (13)	.69	**.10**	**3592.84**
	Distractor template	No distractor	875 (24)	.96 (.01)	–	–	–	–
		Aversive	947 (28)	.95 (.01)	72 (14)	.82	**.09**	**153180**
		Neutral	952 (20)	.96 (.01)	76 (15)	.79	**.11**	**58429.4**
	Target template	No distractor	619 (20)	.97 (.01)	–	–	–	–
		Aversive	657 (26)	.97 (.01)	38 (11)	.53	**.11**	**74.33**
		Neutral	640 (22)	.95 (.01)	21 (8)	.41	**.10**	**6.37**
Experiment 2a	No template	No distractor	821 (27)	.95 (.01)	–	–	–	–
		Aversive	883 (30)	.94 (.02)	61 (13)	.89	**.09**	**38111.05**
		Neutral	893 (32)	.93 (.02)	72 (13)	1.05	**.08**	**2719586**
		Shape	836 (29)	.95 (.01)	15 (13)	.20	**.27**	.62
	Distractor template	No distractor	853 (30)	.95 (.02)	–	–	–	–
		Aversive	877 (30)	.93 (.02)	24 (15)	.29	**.26**	1.39
		Neutral	893 (29)	.93 (.02)	40 (16)	.44	**.20**	**8.55**
		Shape	860 (32)	.94 (.01)	8 (12)	.12	**.31**	.33
Experiment 2b	No template	No distractor	1276 (23)	.97 (.01)	–	–	–	–
		Aversive	1354 (24)	.97 (.01)	78 (16)	.68	**.11**	**28102.15**
		Neutral	1347 (22)	.98 (.01)	71 (18)	.84	**.16**	**559.31**
		Shape	1283 (21)	.98 (.01)	7 (13)	.35	.36	**.32**
	Distractor template	No distractor	1269 (22)	.97 (.01)	–	–	–	–
		Aversive	1313 (22)	.97 (.01)	43 (13)	.46	**.14**	**58.07**
		Neutral	1348 (21)	.97 (.01)	79 (14)	.79	**.09**	**1151335**
		Shape	1280 (22)	.97 (.01)	10 (17)	.09	.44	.44

Raw mean distractor costs (distractor RT minus no distractor RT) are also reported with standard error, standardised effect sizes, and Bayes factors for both the effective inhibition hypothesis (Bayesian prior = −22 ms), and increased capture hypothesis (Bayesian prior = 65 ms). Bayes factors are interpreted on a continuous scale of evidence, though we have highlighted values above 3 and below .33 in bold, which reflect moderate evidence for the experimental and null hypotheses (Wagenmakers et al., 2018).

In contrast, the salient shape distractors in Experiment 2a/2b showed evidence against both inhibition and capture. Bayes factors favoured the null for both hypotheses, suggesting that shapes only caused minor interference and were not suppressed below baseline.

## Template cueing effects

### Experiment 1

**Fig. 2:**
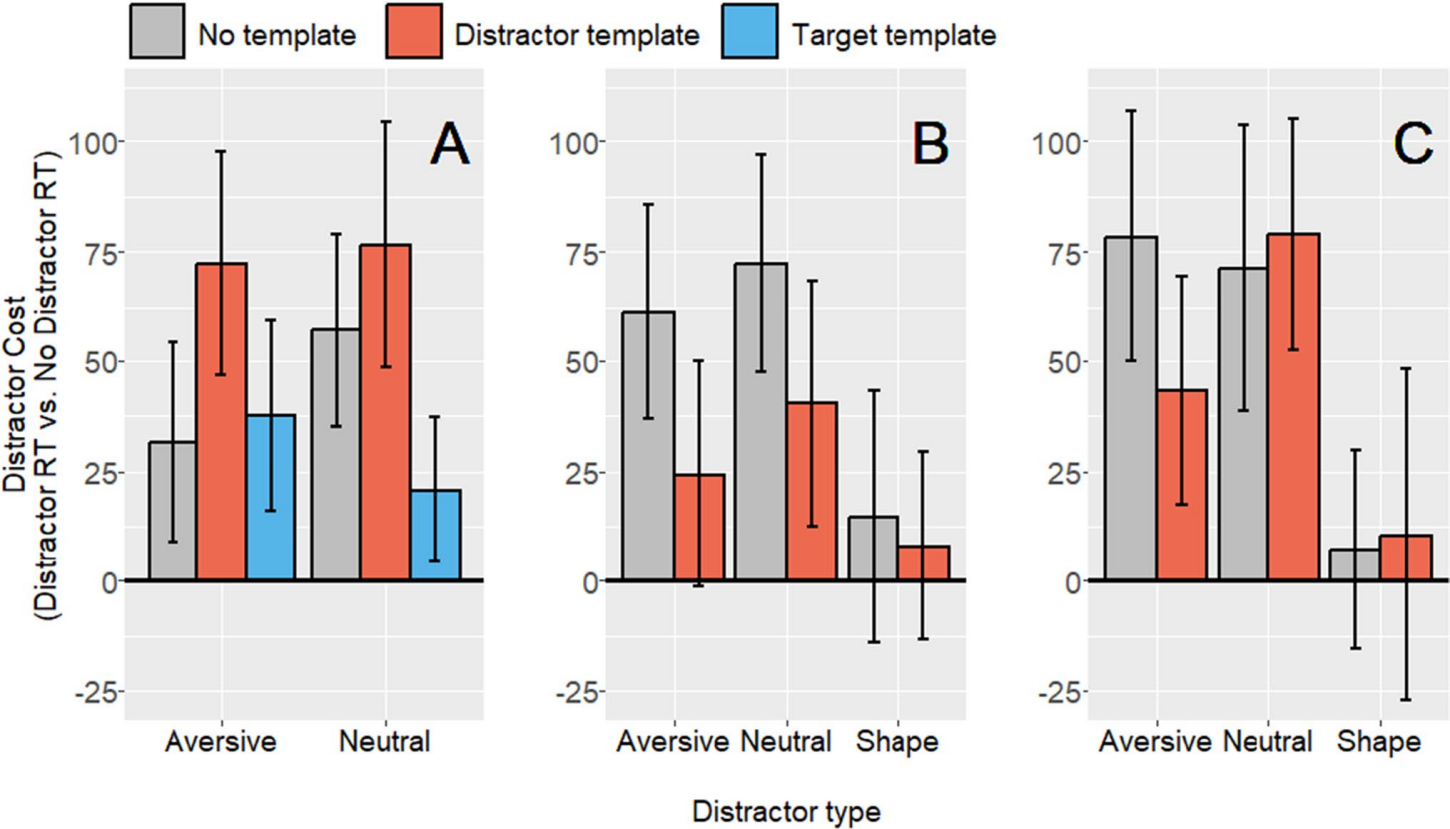
Distractor costs (distractor RT minus no distractor RT) for each distractor type across all template conditions, in **A** Experiment 1; **B** Experiment 2a; and **C** Experiment 2b. Error bars reflect within-subjects 95% confidence intervals (Cousineau, 2005)

The planned Bayesian pairwise comparisons revealed that in Experiment 1 there was evidence of increased attentional capture after cueing the aversive distractor (Table 2; Fig. 2), and anecdotal evidence for increased capture of the neutral distractor (BF = 1–3). Conversely, there was substantial evidence against effective inhibition for both (BF < .33).

**Table 2:** Raw mean template cueing effects (distractor template distractor cost RT minus no template distractor cost RT) are also reported with standard error (*SE*), standardised effect size, and Bayes factors for both the effective inhibition hypothesis (Bayesian prior = −42 ms), and increased capture hypothesis (Bayesian prior = 40 ms)

Experiment	Distractor cost type	Mean difference in distractor cost vs no template (*SE*)	Cohen’s *d*_z_	H_inhibition_ Bayes factor	H_capture_ Bayes factor
Experiment 1 *N* = 40	Aversive	40.73 (18.70)	.34	**.14**	**5.79**
	Neutral	19.26 (18.52)	.16	**.22**	1.09
Experiment 2a *N* = 30	Aversive	−37.06 (19.68)	−.34	**3.47**	**.17**
	Neutral	−31.83 (19.71)	−.30	2.23	**.19**
	Shape	−6.6 (16.61)	−.07	.51	**.29**
Experiment 2b *N* = 50	Aversive	− 34.94 (20.84)	−.24	2.57	**.19**
	Neutral	7.83 (23.69)	.05	.39	.63
	Shape	3.44 (23.39)	.02	.44	.54

Bayes factors were computed with a half-normal distribution centred on zero. Bayes factors are interpreted on a continuous scale of evidence, though we have highlighted in bold values above 3 and below .33, which reflect moderate evidence for the experimental and null hypotheses

Analysis of the target template cueing effects revealed anecdotal evidence against increased capture by the aversive distractor, B_H[0, 40]_ = .52, and moderate evidence against effective inhibition, B_H[0, -42]_ = .28. Whilst for neutral distractors, evidence strongly favoured effective inhibition, B_H[0, -42]_ = 18.65, and went against increased capture, B_H[0, 40]_ = .09.

### Experiments 2a and 2b

In a reversal from Experiment 1, in both Experiment 2a and 2b Bayes factors showed moderate evidence *against* increased capture after cueing the distractor template. In Experiment 2a, there was moderate evidence against an increased capture by both neutral and aversive distractors (BF < .33). In Experiment 2b, there was moderate evidence against increased capture by aversive distractors (BF < .33), but only anecdotal evidence against increased capture by neutral distractors after cueing (BF = .33–1).

Instead, evidence favoured effective inhibition in Experiments 2a and 2b, with moderate evidence of effective inhibition for aversive distractors in 2a (BF > 3), and anecdotal evidence in 2b (BF = 1–3). The pattern was less consistent for neutral distractors, which showed anecdotal evidence in favour of effective inhibition in Experiment 2a (BF = 1–3), but anecdotal evidence favouring the null in Experiment 2b (BF = .33–1).

For the shape distractor condition, distractor template cueing showed anecdotal evidence for both hypotheses, indicating that there was minimal effect of template cueing on distractor processing in either direction.

#### Post hoc Bayesian meta-analysis

We note that the template cueing effects were relatively small, and in the frequentist analysis the interaction in a 2 × 4 ANOVA failed to reach significance for Experiments 2a/2b (*p* > .147). Only in the planned contrasts (see preregistration: osf.io/hv42u), was there evidence of successful inhibition for aversive distractors (one-tailed pairwise comparisons, *p* < .05).

Importantly, a Bayesian analysis of the meta-analytically computed posterior estimate for Experiment 2 revealed strong evidence (BF > 10) of inhibition for aversive distractors, *M*_*diff*_ = -36.06ms, *SE* = 14.31, B_H[0,-42]_ = 10.10, and strong evidence against increased capture, B_H[0,40]_ = .10. Whilst for neutral distractors there was inconclusive evidence of inhibition, *M*_*diff*_ = −15.61, *SE* = 15.15, B_H[0,-42]_ = .90, and moderate evidence against increased capture, B_H[0,40]_ = .19. For the shape distractor, there was anecdotal evidence against effective inhibition, *M*_*diff*_ = 3.23, *SE* = 13.54, B_H[0,-42]_ = .37, as well as moderate evidence against increased capture, B_H[0,40]_ = .27, suggesting no change after cueing.

### Aversive versus neutral distractor contrasts

#### Experiment 1

There was moderate evidence against distractor templates inhibiting aversive more than neutral distractors in Experiment 1, *d*_*z*_ = .18, B_H[0,-42]_ = .21, and inconclusive evidence that they increased capture for aversive versus neutral distractors, B_H[0,40]_ = 1.22.

#### Experiments 2a and 2b

Experiment 2a revealed that there was anecdotal evidence against the aversive distractors being inhibited more, *d*_*z*_ = .04, B_H[0,-42]_ = .55, or resulting in more capture, B_H[0,40]_ = .40, than neutral distractors after distractor template cueing. In comparison, for Experiment 2b there was moderate evidence for greater inhibition of aversive distractors versus neutral distractors, *d*_*z*_ = .42, B_H[0,-42]_ = 3.07, and moderate evidence against increased capture, B_H[0,40]_ = .20.

#### Real-world versus shape distractor contrasts

For Experiment 2a the comparison between the combined real-world distractors, relative to shape distractors, revealed moderate evidence in favour of greater inhibition of real-world distractors, *d*_*z*_ = −.24, B_H[0,-42]_ = 3.01, and moderate evidence against increased capture, B_H[0,40]_ = .13. For Experiment 2b, however, there was inconclusive evidence that the real-world images were inhibited more, *d*_*z*_ = −.17, B_H[0, -42]_ = .96, though there was still moderate evidence against increased capture, B_H[0,40]_ = .21.

Interpreting the pattern across both preregistered contrasts, in Experiment 2a both aversive and neutral distractors were inhibited to a similar level after distractor template cueing, whilst in Experiment 2b there was only evidence that aversive distractors were inhibited, and this was more than neutral distractors.

### Block effects

For all Experiments, the inclusion of block in a repeated-measures ANOVA with cue type and distractor type revealed a significant main effect, with this reflecting a linear decrease in RT across blocks, *p* < .001, η_p_^2^ > .44. There was however no significant interaction between block number and template effect, distractor effect, or their interaction, *p* > .368, η_p_^2^ < .03.

### Accuracy

Analysis of accuracy across experiments using repeated measures ANOVAs revealed no significant interactions, *p* > .057, η_p_^2^ < .08. Thus, observed effects of template on distractor type RT are unlikely to be due to a speed–accuracy trade-off.

## General discussion

Across experiments we found evidence that cued templates-for-rejection could reduce interference from photorealistic images, especially those depicting severe physical injury; however, this was contingent upon task-demands. In Experiment 1, there was evidence favouring increased capture after cueing the aversive distractor; whilst in Experiment 2a/2b, when templates-for-rejection were the only available strategy and there was more time to process the template, evidence now favoured effective inhibition, primarily for aversive distractors. We note, however, that both real-world distractors consistently produced distractor costs, relative to no distractor and low-level salient shape conditions, indicative of some level of processing, regardless of the distractor template.

The current results fit best with models of rational search behaviour, which propose that informative cues are utilized (1) when they cue a strategy that reduces the effort required to complete the search, or provides sufficient benefit relative to the effort expended; (2) there is sufficient motivation to utilize the cued strategy; and (3) the cued strategy (if beneficial and there is sufficient motivation) is accessible to participants based on task-demands (Pauszek & Gibson, 2018). Indeed, it has been found that in visual search with informative cues, that participants will neglect 100% reliable spatial cues if they are only briefly presented or are visually taxing to process (Davis & Gibson, 2012; Gibson & Bryant, 2005; Pauszek & Gibson, 2016). Based on these principals, the design of Experiment 1 is suboptimal for using templates-for-rejection, whilst the changes for Experiment 2a/2b supported this strategy. One key difference was the inclusion of a longer cue presentation time (200 ms versus 400 ms), making it more accessible. This was required likely due to the more varied and multi-faceted stimulus features, relative to the single feature cues typically used.

The stronger evidence in favour of templates-for-rejection in experiments with this longer cue time is unlikely to be due to passive habituation processes, which occur over much longer periods (Snowden et al., 2016) and after repeated stimulus exposures (Bradley et al., 1993; Ferrari et al., 2020). Indeed, the lack of change in the effects across blocks suggests that habituation, or any learning mechanism, played very little role in the current results, which more likely reflects flexible top-down inhibition.

Typically, in studies finding effective inhibition the template type is consistent across a block, making it the only available strategy (Arita et al., 2012). In contrast to previous work and Experiment 2a/2b, in Experiment 1 both target and distractor template trials appeared in a random order within the same block. Evidence has found that when both target and distractor template cues are available, participants will consistently prioritize the target template (Rajsic et al., 2020), likely due to the greater benefit to target detection speed. Indeed, within Experiment 1, we also found faster overall RT on target template trials relative to distractor template trials.^2^ It is likely, therefore, that participants would have prioritized the more beneficial target search strategy across the block, resulting in participants neglecting to effortfully switch to the less efficient template-for-rejection strategy. In support of this possibility, one of the few studies to also present target and distractor template trials within the same block also found no evidence of effective templates-for-rejection (Salahub & Emrich, 2021).

The possible reason that there was actually *increased* aversive distractor costs in Experiment 1, rather than just absence of inhibition, could be due to the established finding that irrelevant threat-related images are less efficiently filtered from VWM (Stout et al., 2013, 2015). Further, failure to exclude these threat-related features from VWM can result in them guiding attention to visually similar distractors in the array (Berggren, 2022). In Experiment 1, participants may have failed to filter the aversive cue from VWM, and without efforts to inhibit these features, they may have guided attention to the matching distractor features. Within the literature, the deficit in filtering irrelevant affective information is especially pronounced in trait anxious individuals. Notably, we also found a positive relationship between higher attentional capture by aversive distractors after cueing and trait anxiety in Experiment 1 (*r* = .45, *p* = .004; see Supplementary Materials 1), consistent with our interpretation.

Regardless of which condition made the template-for-rejection strategy more accessible (i.e., cue duration or removal of competing target template strategy), the data clearly show that Experiment 1 was suboptimal for distractor inhibition, and that the design of Experiment 2a/b facilitated a modest distractor template effect. Thus, the question is not whether individuals have the fundamental mechanism to execute templates-for-rejection to attenuate attentional capture, but rather which conditions enable this strategy to be applied.

The hypothesis that templates-for-rejection are utilized when they provide a benefit to search efficiency can also account for apparent evidence of inhibition of all real-world stimuli in Experiment 2a, but evidence of inhibition only for aversive distractors in 2b. Between these two tasks, the only difference was that 2a required a simple orientation identification response, whilst 2b required a slower long-term memory comparison response.

Between experiments, the distractors were equally disruptive to the search speed in the no template conditions; however, relative to the overall time to identify the target in this condition, the distractors were proportionally more disruptive in Experiment 2a.^3^ It is plausible, therefore, that participants may be more motivated to utilize the template-for-rejection strategy for neutral stimuli when they cause a more noticeable disruption to visual search. Conversely, despite aversive distractors causing a similar cost to search speed in both experiments, participants may have also been motivated to utilize a template-for-rejection to avoid the unpleasant aversive content. Indeed, prior research shows deliberate attentional avoidance of negative emotional content, even without cueing (Armstrong et al., 2014; Schmidt et al., 2012). The finding suggests that affective content may also drive the rational choice to utilize informative cues in visual search, thus extending existing models beyond simple search efficiency (Pauszek & Gibson, 2018).

### Limitations

As noted, the inhibition effects were only modest and did not entirely suppress distractor costs, potentially indicating only a minor driver of attention. From a search efficiency perspective it could be, however, that detection of the target in the six-item array was relatively simple and inhibition was not always required. In future, increasing the distractor interference would likely increase the use of templates-for-rejection. For instance, having multiple exemplars of the cued distractor in the array would increase the utility of suppressing that distractor.

Unexpectedly, aversive stimuli were no more distracting than the neutral stimuli overall. It is, however, not entirely unusual to find an absence of capture by irrelevant photorealistic aversive stimuli in some tasks (e.g., Brown et al., 2020; Qiu et al., 2022; Vogt et al., 2013). Alternatively, though neutral and aversive stimuli were matched in shape/size, other differences in low-level features or familiarity could have obscured the influence of affective content on attention. Importantly, conclusions were based on template cueing effects, rather than the neutral versus aversive comparison.

In future, aversive Pavlovian conditioned stimuli could isolate purely emotional effects (Schmidt et al., 2015). However, multiple affective associations should be explored, not just fear conditioning, as attentional avoidance of disgust-related cues has been found to be stronger (Armstrong et al., 2014), and is more comparable with the injury images used here.

## Conclusion

The current study has revealed the first evidence that individuals can flexibly utilize templates-for-rejection in VWM to attenuate distraction from real-world stimuli. This is, however, only found reliably when the template-for-rejection strategy is optimal, accessible, and participants are motivated to suppress distractor content.

### Supplementary Information

Below is the link to the electronic supplementary material.

Supplementary file1 (PDF 99 KB)

Supplementary file2 (DOCX 31.1 KB)

Supplementary file3 (DOCX 15.6 KB)

## Footnotes

1 For copyright reasons, these specific images cannot be distributed or shared, preview images were made available to reviewers via the Open Science Framework (https://osf.io/nrk7w/?view_only=aa31bf3a33914a98a5a6ca74794b1ed3). Example images are available upon request.

2 The average RT in the target template condition in Experiment 1 was significantly faster than when no template was cued, 638 ms versus 918 ms, *t*(39) = 19.54, *p* < .001, *d*_*z*_ = −3.09, whilst the distractor template provided no such overall benefit (925 ms) relative to when no template was cued, *t*(39) = .73, *p* = .468, *d*_*z*_ = .12.

3 A between-experiments analyses of the neutral distractor cost in the no template cue condition revealed no difference between Experiment 2a and 2b, 72 ms versus 71 ms, *t*(78) = .05, *p* = .956, *d* = .01. However, the comparison of overall search speed in all no template trials between experiments revealed that Experiment 2a’s RT was substantially faster, 858 ms versus 1,315 ms, *t*(78) = 13.39, *p* < .001, *d* = 3.78. Thus, the cost to search speed from the presence of the neutral distractor was relatively larger in Experiment 2a, as a proportion of overall search time. A comparison for aversive distractor costs in the no template trials also revealed no significant difference between experiments, 61 ms versus 78 ms, *t*(78) = .74, *p* = .462, *d* = .17.
