# Supplementary material for: Can templates-for-rejection suppress real-world affective objects in visual search?
Source: Psychon Bull Rev. 2024 Feb 5;31(4):1843–55. doi: 10.3758/s13423-023-02410-2 (PMC11358251; doi:10.3758/s13423-023-02410-2)
Supplement: Supplementary file 1 — Supplementary file1 (PDF 99 KB) [file 13423_2023_2410_MOESM1_ESM.pdf]

Supplementary materials 1

Exploratory Trait Anxiety Correlations

Experiment 1

We conducted four exploratory correlations between the template specific effects on distractor costs (subtracting out No template reaction times) and trait anxiety, for each type of template (Target template, Distractor template) and distractor (neutral, aversive). Bayes factors were computed with a uniform distribution due to no prior knowledge of the potential effect in a similar task, thus making no assumptions about the probability of different observed effect sizes. The largest expected effect was set as $r_z = .31$ reflecting a moderate effect size based on published guidelines (Cohen, 1988), and lowest as zero reflecting a null effect. Both prior and observed effects were fisher's-z transformed to account for non-normality of Pearson's *r* (see Dienes, 2014 for further information). For frequentist analyses, the alpha was Bonferroni corrected for multiple comparisons (corrected $\alpha = .013$).

Experiment 2a and 2b

Based on the evidence of a relationship between trait anxiety and template-for-rejection cueing effects for aversive distractors in Experiment 1, a Bayesian correlation was computed with a half-normal distribution centred on zero, which reflected a directional positive relationship between distractor template cueing effects and trait anxiety. The prior used for this comparison was $r_z = .48$, reflecting the fisher's-z transformed largest Pearson's r coefficient found in Experiment 1 (i.e., $r = .45$).

Results

Relationship with Anxiety

Experiment 1

Exploratory correlations between Distractor template effects and trait anxiety revealed a significant positive relationship when utilising aversive distractor templates, $r = .45$, $p = .004$, $B_{U[0, .31]} = 27.86$ (see Figure 1). The same effect was non-significant for neutral distractors, $r = .06$, $p = .709$, $B_{U[0, .31]} = .62$. The same correlations within the Target template conditions revealed no evidence of a positive relationship, $r < .19$, $p > .249$, $B_{U[0, .31]} < 1.33$.

Experiments 2a and 2b

Correlation analysis in Experiment 2a revealed no evidence favouring a relationship for Distractor template effects, for either aversive: $r = .18$, $p = .345$, $B_{H[0,.48]} = .87$; or neutral distractors: $r = -.05$, $p = .782$, $B_{H[0,.48]} = .30$; Shape: $r = -.04$, $p = .827$, $B_{H[0,.48]} = .32$. For Experiment 2b, against pre-registered expectations in the larger sample, we found no evidence favouring a correlation between the Distractor template effect and trait anxiety for any of the distractors; Aversive: $r = -.05$, $p = .709$, $B_{H[0,.48]} = .22$; Neutral: $r = -.09$, $p = .555$,

$B_{H[0,.48]}$ = .48; Shape: *r* = .14, *p* = .348, $B_{H[0,.48]}$ = .70. With evidence favouring the null across analyses (BF < 1).

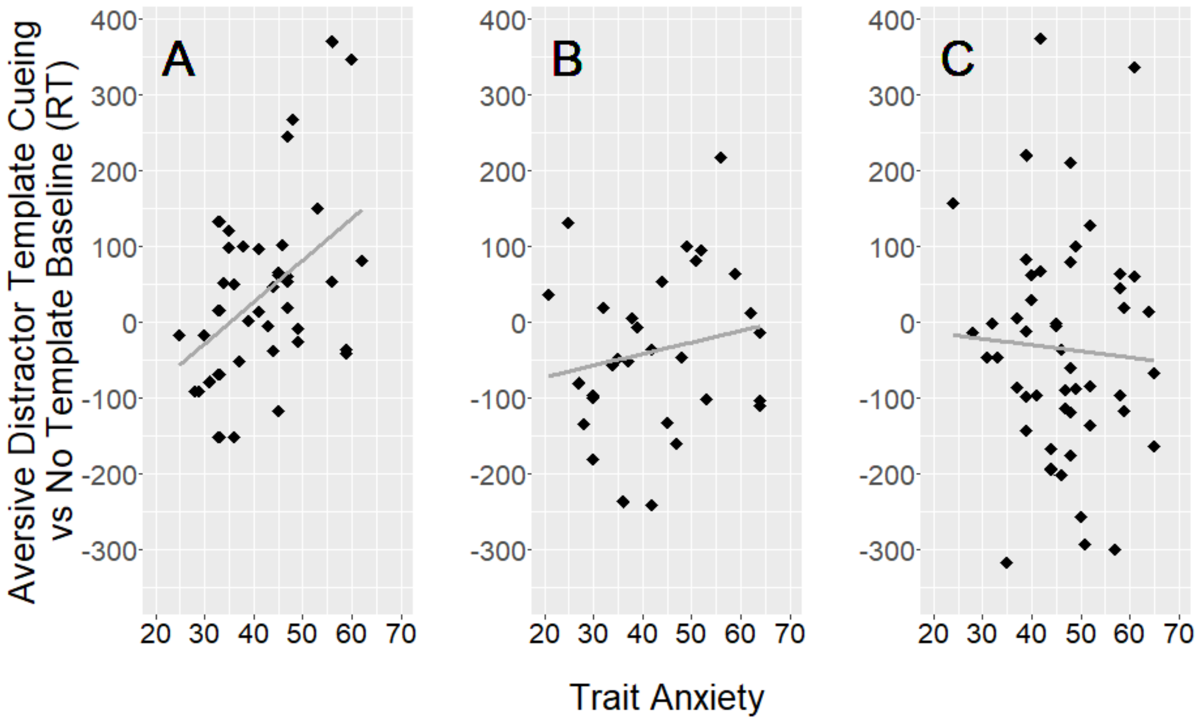

S1 Figure 1. Scatterplots reflecting the relationship between template-for-rejection cueing effects for aversive distractors and trait anxiety across A) Experiment 1; B) Experiment 2a; and C) Experiment 2b. Template-for-rejection effects were computed by subtracting the RT cost when no cue was given from the RT cost when distractor features were cued. Higher scores reflect increased distractor cost after cueing with distractor features.
