# Supplementary material for: Can templates-for-rejection suppress real-world affective objects in visual search?
Source: Psychon Bull Rev. 2024 Feb 5;31(4):1843–55. doi: 10.3758/s13423-023-02410-2 (PMC11358251; doi:10.3758/s13423-023-02410-2)
Supplement: Supplementary file 2 — Supplementary file2 (DOCX 31.1 KB) [file 13423_2023_2410_MOESM2_ESM.docx]

**Supplementary materials 2**

**Frequentist Statistical Analysis**

The dependent variable for the visual search task was RT for correct trials, trials in which responses were incorrect or were over 2500ms or under 100ms were excluded. To explore the overall pattern of results in Experiment 1, an initial 3×3 repeated measures ANOVA with template type (Target template, Distractor template, No template) and distractor type (aversive, neutral, no distractor) as factors was conducted. For Experiment 2a and 2b, due to the change in design, a 2x4 repeated measure ANOVA with template type (Distractor template, No template) and distractor type (aversive, neutral, shape, no distractor) as conditions.

**Planned Contrasts**

***Distractor Cost Effects***

In order to gain a clear interpretable measure of change in RT, we computed distractor cost scores by subtracting the no distractor RT from both the neutral RT and threat-related distractor condition RT, separately. These scores were computed separately for all distractor and template types. If distractors costs were greater than zero this reflected greater interference when the distractor was present versus absent, if they were less than zero this showed evidence of facilitated target detection when the target was present versus absent. To mirror the use of half-normal distributions in the Bayesian analysis, a priori pairwise comparisons were interpreted using one-tailed p-values.

The mean RT scores with the conditions of Experiment 1 were non-normally distributed, to account for this violation of normality computed we bootstrapped 95% confidence intervals from 5000 samples (represented as 95CI_bs_) for distractor costs pairwise comparison (DiCiccio & Efron, 1996). Though Experiment 2a and 2b RTs were normally distributed, confidence intervals were also computed for Experiment 2a and 2b for comparison, as pre-registered. A confidence interval encompassing the value of zero indicates a non-significant effect.

***Template Cueing Effects***

The key comparisons using the distractors costs which tested our specific hypotheses, were between the Distractor template condition and the non-informative No template condition. If distractor costs were significantly lower versus the non-informative No template condition, then this would be evidence that the template-for-rejection was effective at suppressing distractor interference, whilst a significantly higher value would be reflective of counterproductive attentional capture.

**Post Hoc Meta-analysis of Template Cueing Effects.** Within Experiment 2a and 2b, the pattern of results showed some inconsistency despite these Experiments be highly similar and only differing in the target’s identifying feature. In order to assess the cumulative evidence for the observed attenuated attentional capture across Experiment 2a and 2b, we calculated the cumulative effect using the *metafor* R-package (Viechtbauer, 2010). The cumulative effect was calculated using maximum likelihood estimator. Due to the post hoc nature of the analysis, we Bonferroni corrected the alpha for the three comparisons for each distractor type (corrected α = .017).

**Contrasting Specific Distractor Template Effects.**

***Aversive versus Neutral Distractor.*** To make a direct comparison of whether an observed template-for-rejection effect was limited to just affective or neutral stimuli, we made a comparison of the template cueing effects between two distractors. The template cueing effects reflect the difference in distractor cost between the No template and Distractor template conditions – a higher score reflects increased capture, whilst a negative score reflects more effective inhibition of the aversive distractor.

***Real-world versus Abstract Shape Distractor.*** In Experiment 2a and 2b, the inclusion of the salient shape distractor allowed the comparison of Distractor template cueing effects for real-world versus to more conventional salient distractor stimuli. This comparison allowed us to explore whether any observed effective inhibition or increased capture effects were limited to photorealistic stimuli, or generalised to all salient stimuli. The real-world distractor costs were computed by averaging the distractor costs for both neutral and aversive hand distractor stimuli.

**Results**

**Experiment 1**

The initial 3×3 repeated measures ANOVA revealed a significant main effect of distractor type, *F*(2,78) = 28.17, *p* < .001, η^2^_p_ = .42, with both neutral (846ms) and threat-related distractors (841ms) resulting in slower target detection relative to the no distractor condition (794ms). Further, the main effect of template type was also significant, *F*(2,78) = 302.16, *p* < .001, η^2^_p_ = .89 (Huynh-Feldt corrected), with the template-for-rejection resulting in a slower target detection (925ms), relative to a non-informative template (918ms), and target template (638ms). Finally, the interaction between template and distractor types with distractor costs was significant, *F*(4,156) = 4.1, *p* = .003, η^2^_p_ = .10.

***Planned Contrasts***

**Distractor Costs.** Comparison of the RT for each distractor to the No distractor condition, across both template cue conditions, revealed significant attentional capture with longer RTs when both aversive and neutral distractors were present, versus absent (see Table 1.

S2 Table 1. Mean and standard error of reaction time (RT) and accuracy across all conditions of Experiment 1. Raw mean distractor costs (distractor RT minus no distractor RT) are also reported with standard error, standardised effect size, and t-test statistics with one-tailed p-values. Bootstrapped 95% confidence intervals are also reported.

|  | Template | Distractor | Reaction time ms (SE) | Accuracy (SE) | Distractor cost ms (SE) | Distractor cost  Cohen’s d*_z_* | t-value | p-value (one-tailed) | 95% CI_bs_  Lower Bound | 95% CI_bs_  Upper Bound |
| --- | --- | --- | --- | --- | --- | --- | --- | --- | --- | --- |
| Experiment 1 | No template | No distractor | 889 (22) | .96 (.01) | - | - | - | - | - | - |
|  |  | Aversive | 920 (24) | .97 (.01) | 31 (11) | .46 | 2.90 | .003 | 10 | 52 |
|  |  | Neutral | 946 (25) | .95 (.01) | 57 (13) | .69 | 4.36 | < .001 | 32 | 83 |
|  | Distractor template | No distractor | 875 (24) | .96 (.01) | - | - | - | - | - | - |
|  |  | Aversive | 947 (28) | .95 (.01) | 72 (14) | .82 | 5.18 | < .001 | 47 | 99 |
|  |  | Neutral | 952 (20) | .96 (.01) | 76 (15) | .79 | 4.98 | < .001 | 46 | 106 |
|  | Target template | No distractor | 619 (20) | .97 (.01) | - | - | - | - | - | - |
|  |  | Aversive | 657 (26) | .97 (.01) | 38 (11) | .53 | 3.33 | < .001 | 17 | 61 |
|  |  | Neutral | 640 (22) | .95 (.01) | 21 (8) | .41 | 2.57 | .007 | 5 | 37 |

**Template Cueing Effects.** The template cueing effect revealed that there was a significant increase in attentional capture by the aversive distractor after Distractor template cueing, relative to the No template cueing condition, *t*(39) = 2.18, *p =* .018, dz = .34. For the neutral distractor, however, there was no significant increase in capture though it followed the same pattern, *t*(39) = 1.04, *p* = .152, dz = .16.

When comparing the effect of Target template cueing, relative to No template cueing, there was no significant difference between the different types of cue for aversive distractors, *t*(39) = .38, *p* = .353, dz = .06, though there was significantly lower attentional capture for the neutral distractor after cueing with the Target template versus No template, *t*(39) = 2.75, *p* = .004, dz = -.44.

**Experiment 2a**

To assess the interaction between cued template type and distractor type in Experiment 2a and 2b, a 2x4 repeated measures ANOVA was conducted, with cued template type (Distractor template, No template) and distractor type (aversive, neutral, shape, no distractor) as factors. Planned pairwise comparisons were then conducted

There were no violations of normality within the data, therefore, though for comparison to Experiment 1 95% bootstrapped confidence intervals were also computed for both Experiment 2a and 2b.

The initial 2x4 ANOVA revealed a marginally significant effect of template type, *F*(1, 29) = 3.47, *p* = .073, η^2^_p_ = .11, with distractor templates resulting in slightly slower RT overall. There was a significant distractor effect, *F*(3,87) = 12.01, *p* < .001, η^2^_p_ = .29. There was, however, no significant interaction between template cue type and distractor type, *F*(3,87) = 1.83, *p* = .15, η^2^_p_ = .06.

***Planned Contrasts***

**Distractor Costs.** Comparison of each distractor type to the no distractor condition, within each template cue condition revealed that across the no template condition both aversive and neutral distractors resulted in significantly slower RT versus the no distractor condition (see Table 2). When this comparison was, however, conducted in distractor template condition, the aversive distractor cost became non-significant, and the neutral costs was attenuated but remained significant. The shape distractor cost comparison was non-significant in both conditions.

S2 Table 2. Mean and standard error of reaction time (RT) and accuracy across all conditions of Experiment 2a. Raw mean distractor costs (distractor RT minus no distractor RT) are also reported with standard error, standardised effect size, and t-test statistics with one-tailed p-values. Bootstrapped 95% confidence intervals are also reported.

|  | Template | Distractor | Reaction time ms (SE) | Accuracy (SE) | Distractor cost ms (SE) | Distractor cost  Cohen’s d*_z_* | t-value | p-value (one-tailed) | 95% CI_bs_  Lower Bound | 95% CI_bs_  Upper Bound |
| --- | --- | --- | --- | --- | --- | --- | --- | --- | --- | --- |
| Experiment 2a | No template | No distractor | 821 (27) | .95 (.01) | - | - | - | - | - | - |
|  |  | Aversive | 883 (30) | .94 (.02) | 61 (13) | .89 | 4.89 | < .001 | 35 | 85 |
|  |  | Neutral | 893 (32) | .93 (.02) | 72 (13) | 1.05 | 5.72 | < .001 | 48 | 96 |
|  |  | Shape | 836 (29) | .95 (.01) | 15 (13) | .20 | 1.11 | .137 | -11 | 40 |
|  | Distractor template | No distractor | 853 (30) | .95 (.02) | - | - | - | - | - | - |
|  |  | Aversive | 877 (30) | .93 (.02) | 24 (15) | .29 | 1.57 | .063 | -5 | 54 |
|  |  | Neutral | 893 (29) | .93 (.02) | 40 (16) | .44 | 2.47 | .010 | 9 | 74 |
|  |  | Shape | 860 (32) | .94 (.01) | 8 (12) | .12 | .67 | .254 | -15 | 32 |

**Template Cueing Effect.** Comparison of the distractor costs between Distractor template and No template conditions revealed that there was a significant decrease in attentional capture by the aversive distractor after Distractor template cueing, relative to the No template cueing condition, *t*(29) = 1.88, *p =* .035, dz = -.34. For the neutral distractor there was however no significant decrease in capture, though it followed the same pattern, *t*(29) = 1.62, *p* = .059, dz = -.30. For the shape distractor there was also no significant difference in the distractor cost between No template and Distractor template conditions, *t*(29) = .40, *p* = .346, dz = -.07.

Comparison of the aversive Distractor template cueing effects (i.e., No template versus Distractor template distractor costs) to the neutral distractor template cueing effects revealed no significant difference, *t*(29) = .24, *p* = .405, dz = -.04. Comparison of the real-world distractor costs, computed from the combined neutral and aversive costs, however, revealed that the combined Distractor template cueing effects were significantly more negative than the shape distractor cueing effects, *t*(29) = 1.87, *p* = .036, dz = -.34, consistent with greater inhibition of the distractor features.

**Experiment 2b**

The 2x4 ANOVA with RT as the dependent variable revealed no significant difference between template cue types, *F*(1,49) = 2.24, *p* = .141, η^2^_p_ = .04. Though there was a significant difference between distractor types, *F*(3,147) = 21.22, *p* < .001, η^2^_p_ = .30. There was however no significant interaction between the two factors, *F*(3, 147) = 1.56, *p* = .202, η^2^_p_ = .03.

S2 Table 3. Mean and standard error of reaction time (RT) and accuracy across all conditions of Experiment 2b. Raw mean distractor costs (distractor RT minus no distractor RT) are also reported with standard error, standardised effect size, and t-test statistics with one-tailed p-values. Bootstrapped 95% confidence intervals are also reported.

|  | Template | Distractor | Reaction time (SE) | Accuracy (SE) | RT Distractor cost (SE) | Distractor cost  Cohen’s d*_z_* | t-value | p-value (one-tailed) | 95% CI_bs_  Lower Bound | 95% CI_bs_  Upper Bound |
| --- | --- | --- | --- | --- | --- | --- | --- | --- | --- | --- |
| Experiment 2b | No template | No distractor | 1276 (23) | .97 (.01) | - | - | - | - | - | - |
|  |  | Aversive | 1354 (24) | .97 (.01) | 78 (16) | .68 | 4.83 | < .001 | 47 | 109 |
|  |  | Neutral | 1347 (22) | .98 (.01) | 71 (18) | .84 | 3.87 | < .001 | 35 | 108 |
|  |  | Shape | 1283 (21) | .98 (.01) | 7 (13) | .35 | .54 | .294 | -17 | 32 |
|  | Distractor template | No distractor | 1269 (22) | .97 (.01) | - | - | - | - | - | - |
|  |  | Aversive | 1313 (22) | .97 (.01) | 43 (13) | .46 | 3.22 | .001 | 17 | 69 |
|  |  | Neutral | 1348 (21) | .97 (.01) | 79 (14) | .79 | 5.57 | < .001 | 52 | 105 |
|  |  | Shape | 1280 (22) | .97 (.01) | 10 (17) | .09 | .60 | .275 | -23 | 43 |

**Planned Contrasts**

**Template Cueing Effects.** For Experiment 2b, the comparison of the distractor costs between Distractor template and No template conditions revealed that there was a marginally significant decrease in attentional capture by the aversive distractor after Distractor template cueing, relative to the No template cueing condition, *t*(49) = 1.68, *p =* .05, dz = -.24. For the neutral distractor there was however no significant decrease in capture, though it followed the same pattern, *t*(49) = .33, *p* = .371, dz = .05. For the shape distractor there was also no significant difference in the distractor cost between No template and Distractor template conditions, *t*(49) = .14, *p* = .442, dz = .02.

Comparison of the aversive distractor template cueing effects (i.e., No template versus Distractor template distractor costs) to the neutral distractor template cueing effects revealed significantly lower attentional capture for aversive distractors after cueing, *t*(49) = 1.78, *p* = .041, dz = -.25. Comparison of the real-world distractor costs, computed from the combined neutral and aversive distractor costs, to the shape distractor costs revealed no significant difference, *t*(49) = 1.03, *p* = .155, dz = -.15.

**Post Hoc Meta-analysis of Template Cueing Effects**

The post-hoc meta-analysis revealed a significant decrease in attentional capture by aversive distractors after cueing with the Distractor template, *M_diff_ =* -36.06, *SE* = 14.31, *p* = .012, 95CI[-64.10, -8.02]. There was however no significant difference in this comparison for the neutral distractor, *M_diff_ =* -14.18, *SE* = 19.71, *p* = .472, 95CI[-52.81, 24.45], or the shape distractor after distractor cueing, *M_diff_* = -3.23, *SE* = 13.23, *p* = .811, 95CI[-29.78, 23.31]. Bonferroni correction to account for the three post-hoc meta-analytic comparisons resulted in no change to the level of significance (corrected α = .017).

**Accuracy analysis**

For the exact pattern of accuracy, see Tables 1 – 3. In Experiment 1, there was no significant difference across template cue types, *F*(1.22,47.51) = .49, *p* = .524, η^2^_p_ = .01 (Hyunh-Feldt corrected). Though there was a significant difference between distractor types, *F*(1.75, 68.09) = 3.34, *p* = .048, η^2^_p_ = .08 (Hyunh-Feldt corrected), with neutral distractor appearing to result in lower accuracy. The interaction between template type and distractor type was non-significant, *F*(4,156) = 2.35, *p* = .057, η^2^_p_ = .06.

In Experiment 2a, there was a no significant difference between No template and Distractor template cued trials, *F*(1,29) = 2.12, *p* = .156, η^2^_p_ = .07, across the four different distractor types, *F*(3,87) = 2.42, *p* = .071, η^2^_p_ = .08, or the interaction between these factors, *F*(3,87) < .01, *p* = .664, η^2^_p_ = .02.

In Experiment 2b, there was a significant difference between template cue types, *F*(1,49) = 4.53, *p* = .038, η^2^_p_ = .09, with Distractor template cued trials resulting in more errors. When comparing across distractor types there was no significant difference, *F*(3,147) = .575, η^2^_p_ = .01, and the interaction between these variables was also non-significant, *F*(3,147) = .29, *p* = .830, η^2^_p_ = .01.

**References**

DiCiccio, T. J., & Efron, B. (1996). Bootstrap confidence intervals. *Statistical Science, 11*(3), 189-228.

Viechtbauer, W. (2010). Conducting meta-analyses in R with the metafor package. *Journal of Statistical Software,36*, 1–48.
