# Supplementary material for: Can templates-for-rejection suppress real-world affective objects in visual search?
Source: Psychon Bull Rev. 2024 Feb 5;31(4):1843–55. doi: 10.3758/s13423-023-02410-2 (PMC11358251; doi:10.3758/s13423-023-02410-2)
Supplement: Supplementary file 3 — Supplementary file3 (DOCX 15.6 KB) [file 13423_2023_2410_MOESM3_ESM.docx]

**Supplementary materials 3**

S3 Table 1. Raw mean template cueing effects (Distractor template distractor cost RT minus No template distractor cost RT) are also reported with standard error, standardised effect size, and Bayes factors for both the effective inhibition hypothesis (Bayesian prior = -42ms), and increased capture hypothesis (Bayesian prior = 40ms). Bayes factors in this table are computed with a uniform distribution, which do not adjust for probability of small or large effects. Bayes factors are interpreted on a continuous scale of evidence, though values above 3 and below .33 are highlighted in bold, which reflect moderate evidence for the experimental or null hypotheses.

| Experiment | Distractor cost type | Mean difference in distractor cost vs no template (SE) | Cohen’s dz | H_inhibition_  Bayes factor | H_capture_  Bayes factor |
| --- | --- | --- | --- | --- | --- |
| Experiment 1  N = 40 | Aversive | 40.73 (18.70) | .34 | **.18** | **5.90** |
|  | Neutral | 19.26 (18.52) | .16 | **.28** | 1.43 |
| Experiment 2a  N = 30 | Aversive | -37.06 (19.68) | -.34 | **3.94** | **.22** |
|  | Neutral | -31.83 (19.71) | -.30 | 2.80 | **.24** |
|  | Shape | -6.6 (16.61) | -.07 | .68 | .38 |
| Experiment 2b  N = 50 | Aversive | - 34.94 (20.84) | -.24 | 2.97 | **.25** |
|  | Neutral | 7.83 (23.69) | .05 | .53 | .85 |
|  | Shape | 3.44 (23.39) | .02 | .59 | .74 |
